# Mixing Synthetic and Real Images Improves Artificial Intelligence-Based Detection of the Pupil, Iris, and Sclera: A Cross-Domain Validation Study

**DOI:** 10.7759/cureus.109409

**Published:** 2026-05-21

**Authors:** Krishna Keshav, Deepsekhar Das, Sumit Grover, Sahil Agrawal, Avinav Bharti

**Affiliations:** 1 Ophthalmology, All India Institute of Medical Sciences, New Delhi, New Delhi, IND; 2 Ophthalmology, National Cancer Institute, All India Institute of Medical Sciences, New Delhi, Jhajjar Campus, New Delhi, IND; 3 Volunteer, Manthan Eye Healthcare Foundation, Gurgaon, IND; 4 Medical Physics, All India Institute of Medical Sciences, New Delhi, New Delhi, IND

**Keywords:** ai-generated images, ai in ophthalmology, image segmentation, medical image annotation, medical image labeling, mixed datasets, roboflow, validation study

## Abstract

Background

This study aimed to evaluate whether mixing synthetic and real eye images for artificial intelligence (AI) training improves cross-domain segmentation of the sclera, iris, and pupil compared to single-domain datasets.

Methodology

Four Roboflow 3.0 instance segmentation models were trained: (1) 100 AI-generated images, (2) 100 real images, (3) 50:50 mixed, n = 100, and (4) 50:50 mixed, n = 200. All were tested on five AI-generated and five real eye images. Detection accuracy was calculated per structure. Performance was compared using paired t-tests and two-way analysis of variance.

Results

Mixed models eliminated domain-specific failures. Pupil accuracy showed a significant training × test domain interaction (p = 0.003), with single-domain models failing on opposite domains: AI-trained 0% on one real image; real-trained 50% on one AI image. The 100-Mix model achieved 88.5% ± 5.9% pupil accuracy with no failures, with a standard deviation of <6% versus 29.8% for AI-only. Doubling mixed data to 200 images gave no added benefit (p = 0.95).

Conclusions

Hybrid training with 50:50 synthetic-real images achieves robust, domain-stable detection of the pupil, iris, and sclera. Mixed datasets, not larger datasets, are key for clinically deployable ocular AI.

## Introduction

Artificial intelligence (AI) refers to the use of structured computer algorithms to simulate human intelligence. AI models are basically virtual brains, trained in specific tasks, capable of accurately searching, detecting, targeting, or solving a specified problem [1]. In the last few years, its usage has become increasingly popular in the field of medicine [2]. There are multiple studies showing the use of various large language models, which are nothing but AI models, in various domains of medical sciences, from obtaining information to generating informed consent for medical procedures [3-5].

In ophthalmology, identifying and demarcating various parts and anatomical landmarks of an eye is crucial. In this space, AI algorithms have immense potential. By leveraging the imaging-rich nature of ophthalmology and optometry, it is rapidly transforming this specialty, thereby addressing the global burden of multiple ocular diseases. Its ability to analyze complex imaging and volumes of clinical data allows it to serve unprecedentedly in diagnosis, management, and patient outcomes [6].

A typical AI application network comprises an algorithm that is already pretrained on a dataset. The clinical efficiency of any AI model is directly proportional to the robustness of the datasets it is trained on. In the present scenario, for developing AI models in ophthalmology, one of the main hurdles remains the dataset of images. The creation of databases is time-consuming, as is the process of standardizing the acquired images, and obtaining ethical clearance for usage is another rate-limiting step. A possible answer to this problem is using generative AI (GAI) models.

GAI is a class of algorithms capable of creating original content, such as text, images, videos, music, and even engaging conversations. Since its inception, generative AI has evolved exponentially from multiple architectures, especially transformers for language and GANs for images [7]. At present, there are freely available AI models that can generate hyper-realistic images of not just abstract inanimate objects but human anatomical parts, such as eyes, in response to a simple prompt.

However, the use of these AI-generated human eye images in training AI models for ophthalmology has not been studied much. We conducted a study to assess the applicability of AI-generated eye images against smartphone-captured real eye images in the training of AI models.

## Materials and methods

Study design and objective

This was a cross-domain validation study designed to evaluate the comparative efficacy of synthetic, real-world, and hybrid image datasets in training a computer vision model for the segmentation of three ocular structures: sclera, iris, and pupil. Four independent segmentation models were trained under different dataset compositions and evaluated on a common test set comprising both image types.

Synthetic image generation

**Figure 1:**
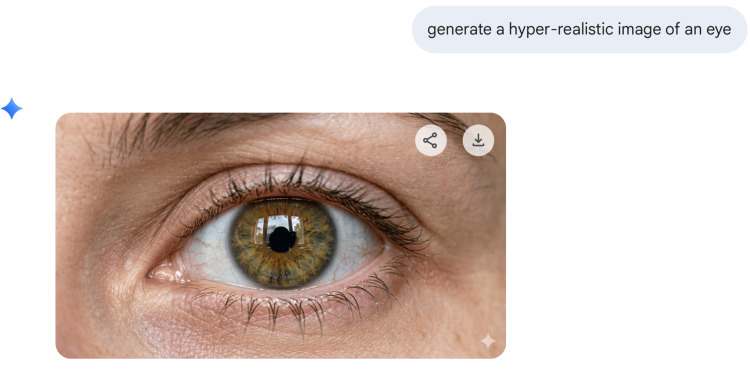
An artificial intelligence-generated image of the eye using Gemini 3.0.

Synthetic eye images were generated using Gemini 3.0 (Google DeepMind), a publicly accessible GAI chatbot. A standardized text prompt, “Generate a hyper-realistic image of an eye,” was used consistently across all iterations to minimize prompt-induced variability. A total of 100 synthetic images were generated to ensure adequate morphological diversity within the artificial image set. This included images of eyes of various races. All generated images depicted an isolated eye without orbital or periorbital pathology (Figure 1).

Clinical data acquisition

**Figure 2:**
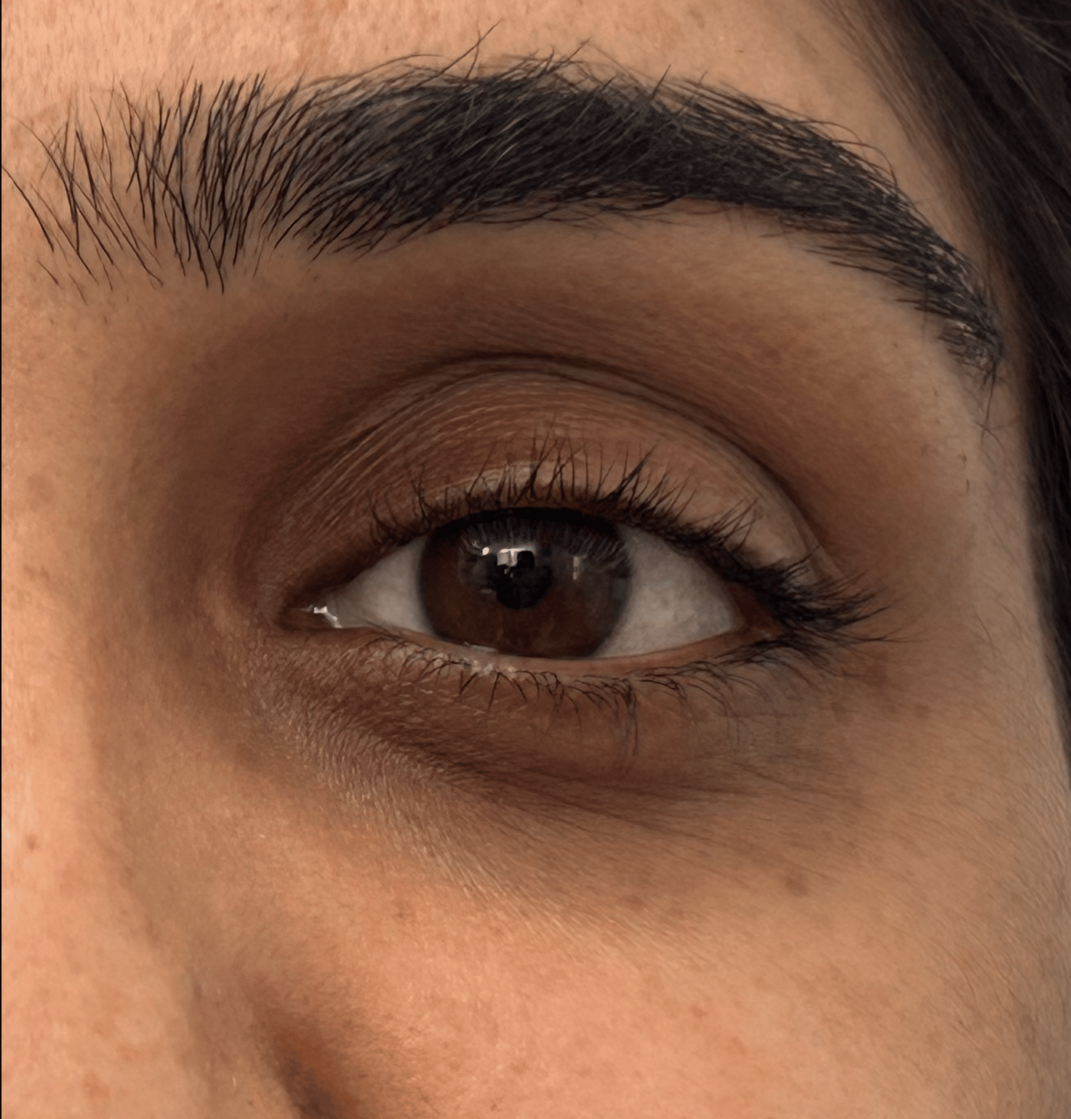
A real image obtained from a patient with refractive error.

Real-world eye images were acquired from patients attending the outpatient department of a tertiary care ophthalmology center in Northern India. Institutional ethical clearance was obtained before data collection (AIIMS Ethics Committee, approval number: AIIMSA5196, dated November 7, 2025), and written informed consent was obtained from all participants. Patients with clinically apparent ocular surface disease, eyelid pathology, or significant anterior segment abnormality were excluded to ensure comparability with the synthetic images. Images were captured using a smartphone camera under standardized lighting conditions. Post-acquisition cropping was applied where necessary to standardize image dimensions, framing, and visual composition relative to the synthetic dataset, while simultaneously removing any identifiable patient features to preserve anonymity. A total of 100 real images were collected (Figure 2).

Image annotation and preprocessing

**Figure 3:**
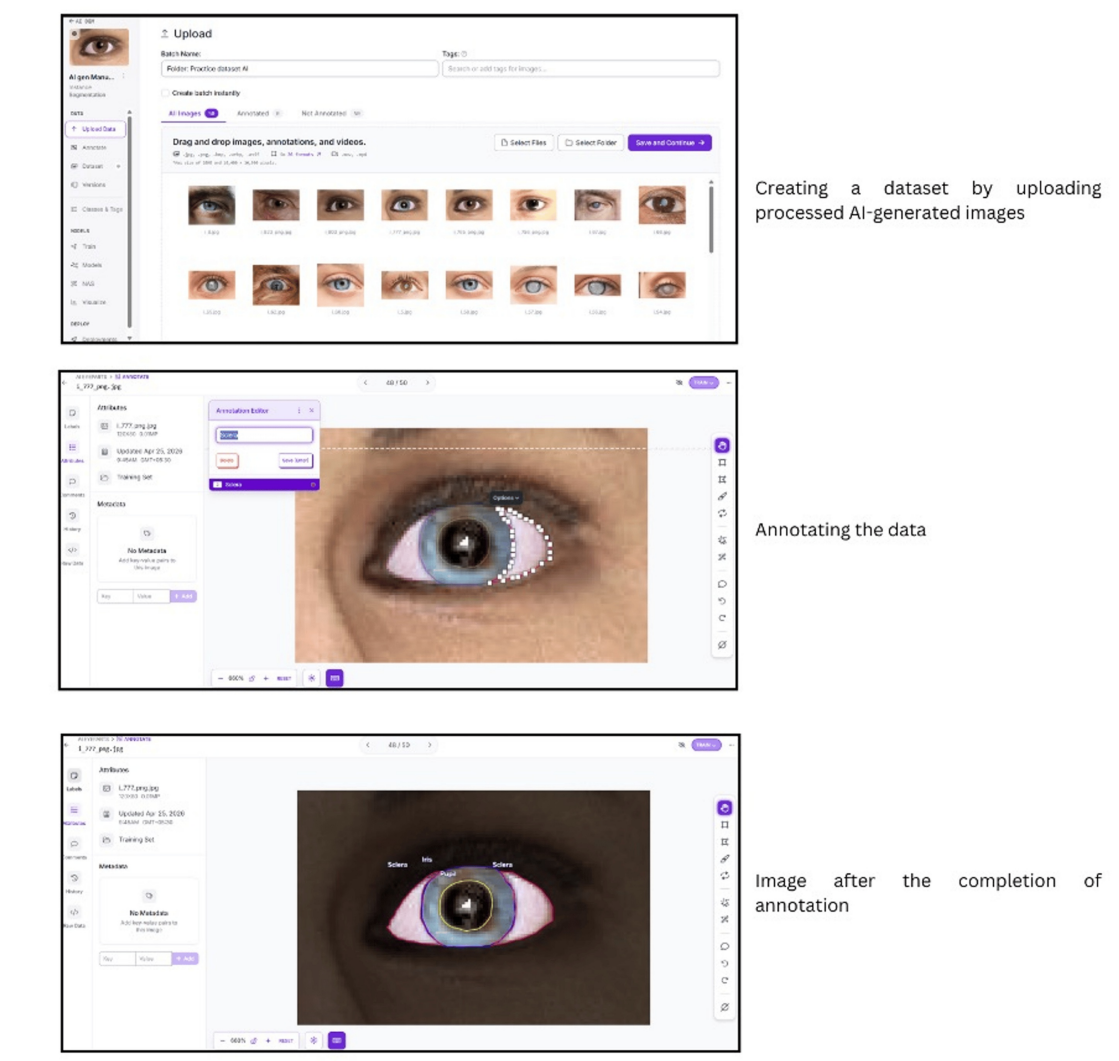
The process of annotation of images.

Both synthetic and real images were uploaded to the Roboflow platform (Roboflow Inc., USA) for annotation and preprocessing. Three classes of interest (COIs) were defined for pixel-level instance segmentation: (1) sclera, the white fibrous outer tunic of the globe; (2) iris, the circular pigmented diaphragm regulating pupillary diameter; and (3) pupil, the central aperture of the iris through which light enters the eye. Annotations were performed manually by trained annotators using Roboflow’s polygon tool to delineate each COI. Annotated masks served as ground truth labels for supervised model training. Standard preprocessing augmentations available within Roboflow (including horizontal flipping, brightness adjustment, and rotation) were applied uniformly across all datasets to enhance generalization (Figure 3).

Model building and training

**Figure 4:**
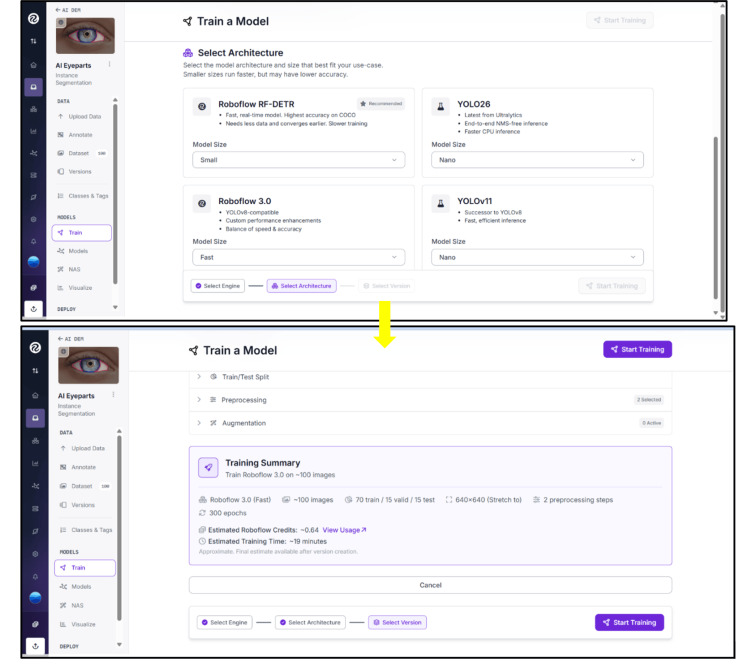
The process of training using Roboflow software.

All segmentation models were developed using the Roboflow 3.0 instance segmentation framework, which employs a YOLOv8-based architecture optimized for object detection and pixel-level segmentation. The intersection of union (IoU) for this model is standardized at 0.5, which gives it an accuracy in mean average precision at 50 values (mAP@50). Four distinct models were trained, each differing only in dataset composition: (1) AI-only model, trained exclusively on 100 AI-generated images; (2) real-only model, trained exclusively on 100 real clinical images; (3) 100-Mix model, trained on a balanced 50:50 mixture of 50 AI-generated and 50 real images (n = 100 total); and (4) 200-Mix model, trained on a balanced 50:50 mixture of 100 AI-generated and 100 real images (n = 200 total). Each model was trained for 300 epochs using the “fast” model size configuration within Roboflow. Preprocessing and augmentation steps were held constant across all four models to ensure that differences in performance could be attributed solely to dataset composition (Figure 4).

Testing protocol and performance evaluation

A dedicated test set comprising 10 images, which were not used in the maturation of the AI model, i.e., five AI-generated and five real, was drawn from the annotated dataset and held out from training. This set was identical across all four model evaluations to enable direct performance comparison. Model performance was quantified as the percentage detection accuracy for each COI per image, calculated as the IoU confidence score returned by the Roboflow inference engine for each annotated structure, compared against the manually annotated ground truth masks. Mean accuracy and standard deviation were computed per structure per model.

Statistical analysis

Statistical analysis was performed using IBM SPSS Statistics version 26.0 (IBM Corp., Armonk, NY, USA). Differences in detection accuracy across the four training conditions were assessed using one-way repeated-measures analysis of variance (ANOVA) with Greenhouse-Geisser correction, where Mauchly’s test indicated violation of sphericity. Region-wise pairwise comparisons (sclera vs. iris, iris vs. pupil, sclera vs. pupil) within each training condition were assessed using paired-samples t-tests. A p-value <0.05 was considered statistically significant.

## Results

In our study, we evaluated the performance of an AI model in recognition of three ocular regions, namely, sclera, iris, and pupil, under four training conditions: AI-generated images only (AI), real images only (Real), a dataset of 100 mixed images (100Mix), and a dataset of 200 mixed images (200Mix). Performance scores for each region were compared across conditions using repeated-measures ANOVA, followed by paired-samples t-tests for region-wise comparisons within each condition (Table 1).

**Table 1:** Confidence of various AI models in detecting various parts of ocular surface when tested on AI-generated and real images. The results of the various models tested using AI-generated and real images. AI = AI-only model; RL = real-only model; 100M = 100-image mixed model; 200M = 200-image mixed model; Sc = sclera; Ir = iris; Pu = pupil; AI = artificial intelligence

	Trained on												
	AI-generated images			Real images			100 mixed images			200 mix images			
Image number	AI-Sc (%)	AI-Ir (%)	AI-Pu (%)	RL-Sc (%)	RL-Ir (%)	RL-Pu (%)	100M-Sc (%)	100M-Ir (%)	100M-Pu (%)	200M-Sc (%)	200M-Ir (%)	200M-Pu (%)	
1	93%	96%	88%	81%	93%	72%	94%	98%	91%	96%	96%	94%	AI-generated image
2	92%	96%	93%	89%	94%	50%	93%	94%	96%	94%	95%	95%	AI-generated image
3	96%	95%	91%	88%	86%	81%	97%	95%	94%	96%	95%	90%	AI-generated image
4	92%	93%	95%	92%	89%	87%	87%	98%	93%	91%	96%	86%	AI-generated image
5	91%	94%	93%	91%	76%	63%	88%	97%	91%	88%	97%	87%	AI-generated image
6	89%	96%	73%	88%	97%	92%	94%	94%	85%	97%	97%	86%	Real image
7	88%	94%	97%	90%	93%	83%	90%	95%	95%	94%	95%	93%	Real image
8	93%	96%	81%	93%	93%	95%	88%	97%	85%	93%	96%	89%	Real image
9	93%	93%	0%	90%	96%	55%	91%	98%	79%	82%	96%	81%	Real image
10	94%	96%	87%	89%	94%	82%	92%	97%	83%	92%	96%	87%	Real image

Sclera recognition scores

Sclera recognition scores did not differ significantly across the four training conditions (F = 1.709, p = 0.210, Greenhouse-Geisser corrected), and the data met the assumption of sphericity (Mauchly’s W = 0.338, p = 0.139). This indicates that the model’s ability to recognize the scleral region remained stable and consistent regardless of whether it was trained on AI-generated, real, or mixed image datasets.

Iris recognition scores

Iris recognition scores showed a statistically significant difference across the four training conditions (F = 5.169, p = 0.039, Greenhouse-Geisser corrected; sphericity violated: Mauchly’s W = 0.032, p < 0.001), indicating that the composition of the training dataset meaningfully influenced how well the model recognized the iris region.

Pairwise comparisons revealed that sclera scores were significantly higher than iris scores in the AI-only (t = −3.562, p = 0.006), 100Mix (t = −3.642, p = 0.005), and 200Mix (t = −2.413, p = 0.039) conditions, suggesting that the model performed better on sclera than iris across most training configurations. In the Real image condition, however, sclera and iris scores were comparable (t = −0.834, p = 0.426).

When iris scores were compared against pupil scores, significant differences emerged in the 100Mix (t = 3.398, p = 0.008) and 200Mix (t = 4.706, p = 0.001) conditions, with iris scores notably higher than pupil scores in mixed-training scenarios. No significant difference was found between iris and pupil scores in the AI-only condition (t = 1.676, p = 0.128).

Pupil recognition scores

Pupil recognition scores did not show a statistically significant difference across training conditions after Greenhouse-Geisser correction (F = 1.984, p = 0.175; sphericity violated: Mauchly’s W = 0.049, p < 0.001), suggesting the model’s pupil recognition performance was relatively unaffected by the training dataset composition.

Nonetheless, sclera scores were significantly higher than pupil scores when the model was trained on real images (t = 2.720, p = 0.024) and in the 200Mix condition (t = 3.188, p = 0.011), whereas this difference was not significant in the AI-only (t = 1.327, p = 0.217) or 100Mix (t = 1.095, p = 0.302) conditions.

Iris scores were significantly higher than pupil scores in the Real condition (t = 3.021, p = 0.014), 100Mix (t = 3.398, p = 0.008), and 200Mix (t = 4.706, p = 0.001), but not in the AI-only condition (t = 1.676, p = 0.128) (Table 2).

**Table 2:** Summary of statistical comparisons across training conditions and ocular structures. Values are the mean detection accuracy (%). RM-ANOVA with Greenhouse–Geisser correction used for between-condition comparisons; paired-samples t-tests used for region-wise comparisons. Significance level: α = 0.05. *: p < 0.05; **: p < 0.01. RM-ANOVA = repeated-measures analysis of variance; AI = artificial intelligence

Comparison	Test and df	Test statistic	P-value	Significant? (α = 0.05)	Direction of effect
Sclera: across 4 conditions (RM-ANOVA)	F(1.29, 11.6)	F = 1.709	0.210	No	—
Iris: across 4 conditions (RM-ANOVA)	F(1.01, 9.08)	F = 5.169	0.039*	Yes	Composition affects iris recognition
Pupil: across 4 conditions (RM-ANOVA)	F(1.09, 9.82)	F = 1.984	0.175	No	—
Sclera vs. iris — AI-only (paired t)	t(9)	t = −3.562	0.006**	Yes	Sclera > iris
Sclera vs. iris — real-only (paired t)	t(9)	t = −0.834	0.426	No	Comparable
Sclera vs. iris — 100-Mix (paired t)	t(9)	t = −3.642	0.005**	Yes	Sclera > iris
Sclera vs. iris — 200-Mix (paired t)	t(9)	t = −2.413	0.039*	Yes	Sclera > iris
Iris vs. pupil — AI-only (paired t)	t(9)	t = 1.676	0.128	No	—
Iris vs. pupil — Real-only (paired t)	t(9)	t = 3.021	0.014*	Yes	Iris > pupil
Iris vs. pupil — 100-Mix (paired t)	t(9)	t = 3.398	0.008**	Yes	Iris > pupil
Iris vs. pupil — 200-Mix (paired t)	t(9)	t = 4.706	0.001**	Yes	Iris > pupil
Sclera vs. pupil — AI-only (paired t)	t(9)	t = 1.327	0.217	No	—
Sclera vs. pupil — Real-only (paired t)	t(9)	t = 2.720	0.024*	Yes	Sclera > pupil
Sclera vs. pupil — 100-Mix (paired t)	t(9)	t = 1.095	0.302	No	—
Sclera vs. pupil — 200-Mix (paired t)	t(9)	t = 3.188	0.011*	Yes	Sclera > pupil
100-Mix vs. 200-Mix: pupil accuracy (paired t)	t(9)	t ≈ 0.064	0.95	No	No benefit from doubling data

## Discussion

The usual steps of the deep learning process involve first identifying datasets, followed by the formulation of the database. The datasets are then divided into training, validation, and testing datasets. For training an AI model in image feature identification, areas in the images are first labelled, and the AI model is trained on them. The process is popularly known as annotation. When an image is fed into any AI model, it divides the image into regions at the pixel level, a process known as segmentation. To obtain a robust AI model, the model, the set of data, the annotated dataset, and the segmentation all need to be of the highest quality [8-10].

In 2022, Beeche et al. published the Super U-Net architecture, a convolutional neural network capable of medical image segmentation. The model was tested to segment retinal vessels, gastrointestinal polyps, and skin lesions on several image types. The model showed significant success for segmenting retinal vessels, pediatric retinal vessels, gastrointestinal polyps, and skin lesions [11,12].

Similarly, multiple studies have been conducted in the past, wherein images of various investigative modalities, namely, CT images, fundus images, and anterior-segment optical coherence tomography, have been used to train AI models. However, there are no studies on the use of AI-generated images in training AI models, as in our study [12-14].

The Artificial Intelligence and Machine Learning in Ocular Oncology, Retinoblastoma (ArMOR) study was published in 2025, where the authors tested the accuracy of a trained AI and machine learning model in the diagnosis and grouping of intraocular retinoblastoma (iRB) based on the international classification of retinoblastoma (ICRB). The results suggested that expanding image datasets, as well as testing and retesting AI models, helps in improving the accuracy of the AI model [15].

In our study, we evaluated how different training data compositions, i.e., AI-generated, real, and mixed datasets, affect the performance of a recognition model across the three ocular regions: sclera, iris, and pupil. We noted that there was not much improvement between the 100 mixed dataset and the 200 mixed dataset. Overall, the findings indicate the model performance is region-dependent and differentially influenced by the nature of the training data.

Performance of sclera recognition showed the highest accuracy against all the provided datasets, pupil demonstrated low accuracy but was consistent across the datasets; while iris recognition was significantly affected by the training dataset composition, highlighting iris as more complex, as it contains patterns, textures, and variations that are difficult for an AI-generated dataset. Pupil recognition was similar to sclera, as it did not show variation across training conditions. However, it showed lower accuracy than both sclera and iris in several comparisons.

Statistical analysis using repeated-measures ANOVA confirmed the observation that sclera and pupil recognition scores remained stable across all training conditions, while iris recognition varied across different datasets. It is also the area where training data had some impact. Pairwise comparisons make this clearer, as it shows sclera repeatedly outperforming iris across most training conditions, and this gap is reduced with the inclusion of real clinical images. Similarly, iris scores were greater than pupil scores in mixed and real training scenarios, but not when models were trained on AI-generated data, implying that exposure to real-world image diversity benefits the structurally complex ocular regions.

Overall, these results indicate that dataset composition has a region-specific impact, with the greatest accuracy observed in iris recognition. While sclera and pupil recognition remain relatively stable across different training datasets, iris recognition shows significant inclusion of real and mixed data, highlighting the need for high-quality and diverse datasets when modelling complex ocular structures. To utilize and grasp the complete potential of AI, a continuous, sustained multidisciplinary collaboration between a varied set of specialty experts, such as clinicians, data scientists, ethicists, and policymakers, is essential. A rigorous validation process, transparency in algorithm development, and strong ethical oversight are equally important to mitigate risks such as bias, data misuse, and unequal access.

Several limitations of the present study must be acknowledged. First, the sample size was small, with each model trained and evaluated on a maximum of 200 images, which may limit statistical power and the generalizability of the findings. Second, all real images were acquired from a single tertiary care center in Northern India, introducing potential demographic and equipment-related bias that may affect generalizability to other populations and imaging contexts. Third, the synthetic images were generated using a single generative model (Gemini 3.0) with a fixed prompt, which constrains the morphological diversity of the synthetic subset. Fourth, no correction for multiple comparisons was applied to the pairwise t-tests, increasing the risk of Type I error. Therefore, results should be interpreted with appropriate caution. Finally, the study did not include patients with ocular pathology, and the performance of these models on eyes with disease has not been evaluated.

## Conclusions

The findings of this study demonstrate that training dataset composition has a differential and region-specific impact on AI-based ocular segmentation performance. Sclera recognition was robust across all four training conditions, maintaining consistent accuracy regardless of whether models were trained on synthetic, real, or mixed data. Pupil recognition was similarly stable, though it consistently exhibited lower accuracy than the sclera. Iris recognition, in contrast, was significantly influenced by the composition of the training data, with performance improving markedly when real clinical images were incorporated; this reflects the greater anatomical complexity, textural heterogeneity, and inter-individual variation inherent to the iris that synthetic images alone cannot adequately represent. Critically, a balanced 50:50 hybrid dataset of 100 images achieved performance comparable to a doubled mixed dataset of 200 images, indicating that dataset composition, not dataset size, is the primary determinant of model robustness in this context. These results support the use of hybrid synthetic-real training strategies as a pragmatic and resource-efficient approach to developing clinically deployable ocular AI, particularly in settings where large annotated clinical datasets are difficult to obtain. While AI-generated images serve as a useful supplementary data source, they are insufficient as standalone training material for anatomically complex structures such as the iris. Future work should focus on the development of larger, demographically diverse, and ethically sourced datasets, as well as validation of these models in pathological eyes and across different imaging modalities, to establish the clinical utility of this approach more broadly.
